# Construction and application of a co-expression network in *Mycobacterium tuberculosis*

**DOI:** 10.1038/srep28422

**Published:** 2016-06-22

**Authors:** Jun Jiang, Xian Sun, Wei Wu, Li Li, Hai Wu, Lu Zhang, Guohua Yu, Yao Li

**Affiliations:** 1 State Key Laboratory of Genetic Engineering, Shanghai Engineering Research Center of Industrial Microorganisms, School of Life Sciences, Fudan University, Shanghai 200438, China

## Abstract

Because of its high pathogenicity and infectivity, tuberculosis is a serious threat to human health. Some information about the functions of the genes in Mycobacterium tuberculosis genome was currently available, but it was not enough to explore transcriptional regulatory mechanisms. Here, we applied the WGCNA (Weighted Gene Correlation Network Analysis) algorithm to mine pooled microarray datasets for the *M. tuberculosis* H37Rv strain. We constructed a co-expression network that was subdivided into 78 co-expression gene modules. The different response to two kinds of vitro models (a constant 0.2% oxygen hypoxia model and a Wayne model) were explained based on these modules. We identified potential transcription factors based on high Pearson’s correlation coefficients between the modules and genes. Three modules that may be associated with hypoxic stimulation were identified, and their potential transcription factors were predicted. In the validation experiment, we determined the expression levels of genes in the modules under hypoxic condition and under overexpression of potential transcription factors (*Rv0081*, *furA* (*Rv1909c*), *Rv0324*, *Rv3334*, and *Rv3833*). The experimental results showed that the three identified modules related to hypoxia and that the overexpression of transcription factors could significantly change the expression levels of genes in the corresponding modules.

*Mycobacterium tuberculosis* (MTB) is a pathogenic bacterium that causes tuberculosis. MTB infects about a third of the global population and leads to more than two million deaths each year^1^. Because of latency and drug-resistance, MTB can survive in almost all environments. The functions of some essential MTB genes are still not well known, and the regulatory mechanisms also need to be further investigated. In recent years, biochip technologies have developed and cumulative MTB data (microarray data, genome sequence data, and CHIP-Seq data) are now publicly available, which has promoted the understanding of transcriptional regulatory mechanisms in this bacterium. We studied the MTB transcriptional regulation networks reported previously^2^,^3^,^4^,^5^,^6^,^7^ and identified some limitations. First, estimated gene expressional levels are semi-quantitative in microarray analysis. So false positive result may be encountered. Second, in reported regulatory networks, the complexity of transcriptional regulatory mechanisms were unable to reveal. For example, a particular gene might be regulated by different transcription factors (TFs) in different stress situations. Third, because gene numbers in the reported networks varied from 900 to 3000, they cannot be considered as a global regulatory network.

The authors of the gene expression datasets with Gene Expression Omnibus (GEO) accession numbers GSE8786^8^ and GSE9331^9^ proposed different functions about *DosR* (also known as *DevR*). As we’ve known, *DosR* is a transcriptional regulator that forms part of a two component system. Voskuil *et al*. concluded that the respiration-limited environment of the oxygen-depleted non-replicating persistent model recreated at least one fundamental factor (*DosR*) for which the MTB genome encodes a decisive adaptive program. Rustad *et al*. showed that a *DosR* deletion mutant entered bacteriostasis in response to *in vitro* hypoxia with only a relatively mild decrease in viability, and in the murine infection model, the phenotype of the mutant was indistinguishable from that of the parent strain. The results of Rustad *et al*. suggested that additional genes may be essential for entry into and maintenance of bacteriostasis. This controversy has not yet been successfully explained.

Here we present the results of an extensive study of the MTB transcriptional regulation network. We build a dataset containing 3411 genes and their expression profiles from 303 microarrays (see Supplementary Data for details:data_303 file), and then clustered the genes into 78 co-expressed modules. To address the *DosR* functional controversy, we performed a time-course analysis at the module level. We also identified some modules related to hypoxia and predict the potential TFs involved. We conducted a validation experiment to confirm the accuracy of the bioinformatic predictions.

## Results

### Construction and analysis of gene co-expression network

MTB microarrays contained some conditions, which were hypoxia, intracellular, infected mouse model, and DosR mutations. We only selected 70 biochips from four datasets, which contained these conditions. Also these biochips had high quality. To reduce the possible data bias, we mixed data for other conditions into our dataset, which ultimately comprised 303 microarrays and 3411 genes that represented 85% of the MTB genome. To ensure consistency of our analysis results, H37Rv was the only experimental strain chosen.

**Figure 1:**
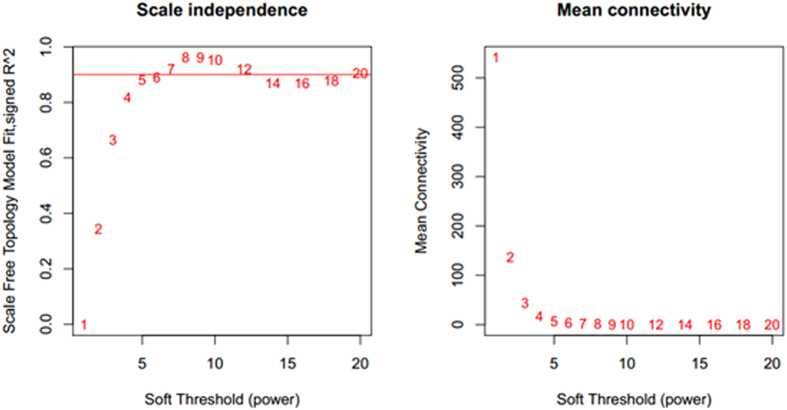
Determination of parameter *β* of the adjacency function in the weighted gene correlation network analysis (WGCNA) algorithm. The adjacency function was weighted by the power of the correlation data between different genes; i.e., *a*_*ij*_ = (*S*_*ij*_, *β*) = |*S*_*ij*_|^*β*^. The weighted parameter *β* in the formula was determined by the scale-free network law, which means that the probability (*p*) that a node is connected with *k* other nodes (*p*(*k*)) satisfies the criterion that the co-efficiency of log(*k*) and log(*p*(*k*)) is at least 0.8. To ensure that the average connectivity of the network is smooth, we chose *β* = 5 based on the diagnosis chart.

The co-expression network was constructed using the WGCNA^10^,^11^ package in R software. The results of the parameter analysis are shown in Fig. 1. After determining the optimal parameter (*β* = 5), the WGCNA algorithm was used to transfer the correlation coefficient between genes into the adjacent coefficient. Then, the dissimilarity of the topological overlap matrix was calculated based on the adjacent coefficient. Using the calculated dissimilarity, we carried out hierarchical analysis by agglomerative hierarchical clustering, also known as the bottom-up method. Other assumptions that we made were: (i) distances between different classes were measured by the average connectivity; and (ii) there should be at least 10 genes in each gene module. (We had tried to put this threshold smaller (<10). But we found these small modules were no biological significance).

**Figure 2:**
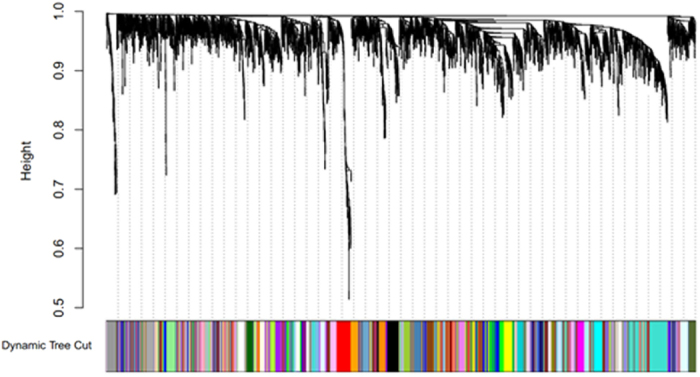
Construction of the gene co-expression network. Each colour represents a certain gene module. There should be at least 10 genes in each gene module.

**Figure 3:**
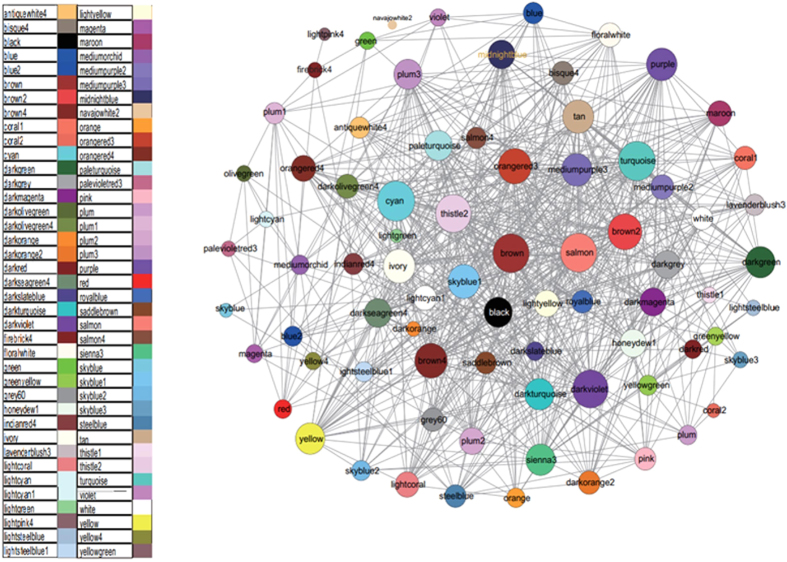
Relationships among the gene modules. Two gene modules are connected if the correlation between them, which takes into account the correlation coefficient between each particular gene and these two modules, is greater than a selected threshold. Different modules are shown in different colours and different sizes. A larger size indicates more connections with other modules; a smaller size indicates fewer connections.

Based on these assumptions, we obtained 78 gene modules as shown in Fig. 2. We got 78 gene modules by the function cutreeDynamic in WGCNA package. We have chosen the soft thresholding power 5, a relatively large minimum module size of 10, and a medium sensitivity (deepSplit = 2) to splits cluster. By the Pearson correlation coefficient between modules, we constructed the network. When the absolute value of correlation was more than 0.45, we would link two modules. The network was shown as in Fig. 3. Gene list information is in module-info supplementary.

To determine the reliability of analysis results, we chose the target genes of two well-studied TFs (*DosR* and *KstR*)^5^ as the testing gene sets. A Fisher’s exact test was used to assess the significance of the correlations between the two testing gene sets and our gene modules. Table 1 shows that the red and ivory modules had significant correlations with target *DosR* and *KstR* gene sets, respectively. Our dataset included a large number of microarrays on hypoxia, intracellular, infected mouse model, and *DosR* mutation conditions. To exclude biased results, we constructed a new co-expression network with 233 microarrays without latency conditions and still obtained significant correlations. These results indicate that the co-expression network is capable of providing accurate predictions about the regulatory relationships.

We also obtained useful information about gene function from the co-expression network. First, when related genes under different experimental conditions clustered into one module, the results can be used to analyse high-throughput experimental data (e.g., transcription profiles) and to identify potential functional genes. Second, the subnetworks of some interesting modules where the nodes were not modules but genes, can be constructed. Genes with high connectivity generally have complicated regulatory mechanisms or specialised functions. Third, by calculating the Pearson correlation coefficient between every gene and a certain gene module, the relationship between that module and the genes outside that module can be estimated. Fourth, based on the correlation information between modules, a connectivity network that provides more understanding about the situation of every module can be constructed. The relationships among gene modules are presented in Fig. 3. Fifth, potential motifs in a certain gene module can be confirmed using the MEME^12^ software. The motif-based sequences are listed in the supplementary materials (motif information file).

### Enrichment analysis of gene modules

To study the potential functions of the gene modules, we performed module enrichment analysis using three well-known annotation databases: GO, KEGG, and the TF gene set in testing dataset. The GO and KEGG functional annotations of the MTB genes were assigned from homology comparisons; therefore, the results may not be very accurate. However, from the overall trend, we considered that these transcriptional regulatory mechanisms tend to be similar to homology comparisons. In addition, the regulation network constructed by Sanz *et al*.^5^ contained a certain proportion of the gene connectivities from experiments or microarray data. Therefore, some target genes regulated by the same TFs and were generally highly coordinated, despite the possibility of false positive results.

To further ensure the reliability of our results, we conducted a statistical comparison between the modules and the gene sets mentioned in *Annotation databases*, as well as between the random permutation modules and the gene sets in *testing dataset*. We applied fisher exact test and student test to do enrichment analysis and significant analysis. As showed in Table 2, the gene modules contained many more genes in TF target gene sets than did the random modules. The target gene sets regulated by the special regulation factor and enriched in our gene modules are shown in Table 3. However, it should be noted that the gene module regulated by a precise regulation factor does not necessarily contain this regulation factor. Indeed, a specified gene module either may not contain any regulation factors or may contain several regulation factors. When a module contains numerous regulation factors, this situation must be analysed under different circumstances. Such a situation may be explained by the complicated transcription regulation mechanisms that bacteria may use to regulate specific gene modules in response to different growth situations and environments. Genes in a modules had similar expression trend in different conditions, although these genes would be regulated by different TFs in various conditions. We identified several highly correlated regulatory factors in our results. In the subsequent analysis, we used the Pearson correlation between gene modules and regulation factors to filter the potential regulation factors under certain circumstances. The possible gene-module-related physiological functions and metabolic pathways for the genes in the KEGG and GO datasets that were enriched in different gene modules are listed in Table 4. (We thought these genes in the same module would have similar functions. So gene functions also could be identified. For example, we predicted the “red” module may be related to hypoxic stress. In validation, we also proved this prediction. So these genes in “red” module had highly possible function about stress to hypoxia.)

### Analysis of two hypoxic models at the module level

The hypoxic model has been used to study the MTB dormant mechanism, and two kinds of hypoxic models have been used in many biochips designed to mimic low-oxygen stress. One model had a constant concentration of hypoxia environment in the chemostat (the typical oxygen concentration was 0.2%). The other was the Wayne growth model, where the experiment was conducted in a hermetically sealed tube over a long time period, so that the oxygen concentration gradually decreased.

Voskuil *et al*.^8^ and Rustad *et al*.^9^ provided time-course data of two hypoxic models (GSE8786 in Wayne and GSE9331 in constant concentration). These researchers conducted approximate comparative analyses for differences and similarities in gene expressions, and studied the *DosR* regulator and a few genes with functions that were well known in these conditions. The experimental results obtained in these two studies lead to different conclusions about *DosR*.

**Figure 4:**
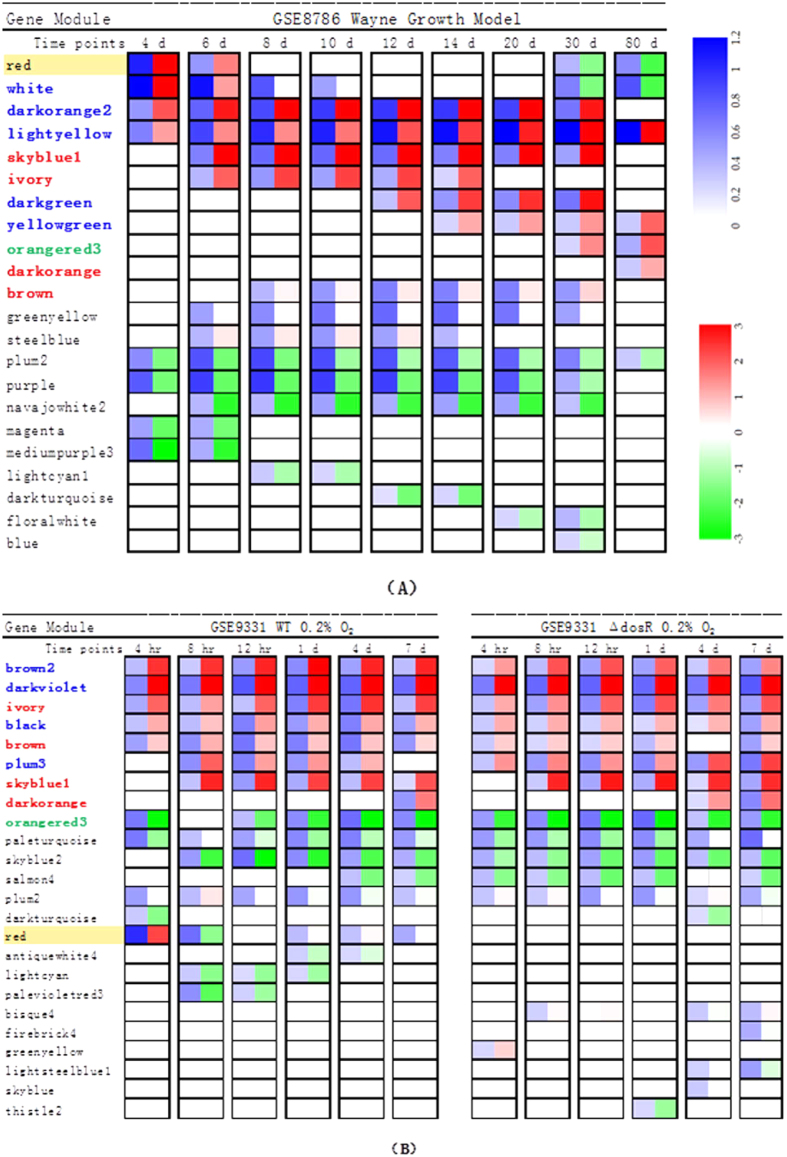
Gene modules with significant change. (**A**) Gene modules with significant change in GSE8786. (**B**) Gene modules with significant change in GSE9331. A modified NetReSFun algorithm was used to determine whether there was a significant change at a certain time. Modules with significant changes (≥0.22) are shown by rectangles: blue (left) indicates the saturation revealed the log converted *Z*-value of the significant change of this module; blue or green (right) indicates this gene module was up- or down-regulated, respectively. A colour gradient map is shown to the right of (**A**). The red module contains most of the DosR regulon and is highlighted in yellow. Modules in a red font were up-regulated in both datasets; green font indicates modules that were regulated in different directions; and blue font indicates modules that were significantly up-regulated and were unique to the dataset they belonged to.

To replay and explain *DosR* functional controversy, we identified the gene modules and the gene expression changes at certain time points using the NetReSFun^2^,^4^ algorithm. The results are shown in Fig. 4. For the Wayne model in GSE8786, the *DosR* regulator (red module) was conspicuously up-regulated at day 4 and day 6, and then plateaued without further fluctuation until it was down-regulated at day 30. These results correlated well with the results of the original study. For the wild-type MTB bacteria under the constant hypoxia model in GSE9331, the *DosR* regulator was significantly up-regulated at the 4-hour time point compared with the starting point (0-hour point), and then notably down-regulated at the 8-hour time point. The expression level of *DosR* then reverted to the initial value after one day (24-hour point), which is in line with the original results of Rustad *et al*. However, the expression of the *DosR* regulator was not altered in the *DosR* mutant strains. Thus, the expression trends of other modules along the time course were similar in the wild-type and mutant strains. Analysis of the results showed that the number of unregulated genes (almost of *DosR* regulon family), which eventually reached about 230, gradually stabilised with time in the wild-type as well as in the *DosR* mutant strains, suggesting that the *DosR* mutation does not affect the number of varying genes. This finding was also consistent with our module analysis, indicating that our analysis method is viable for processing new datasets.

By comparing the significantly up-regulated gene modules in the two datasets (GSE8786 and GSE9331), we attempted to explain the divergence of MTB transcriptional responses to the two hypoxia models. Figure 4 shows that except for the red module (*DosR* regulator), four gene modules (skyblue1, ivory, dark orange, and brown with the red bold marker) in the two datasets were significantly up-regulated, which represented a common adaption of MTB in the two hypoxia models. Especially in GSE8786, the expressions of five modules (white, dark orange2, light yellow, dark green, and yellow-green) were distinctly increased. In GSE9331, four specific gene modules (brown2, dark violet, black, and plum3 with the blue bold marker) were up-regulated. The module orangered3 (green bold marker) showed opposing expression trends in the two datasets. These gene modules may provide clues as to how MTB adjusts to the different hypoxia models.

**Figure 5:**
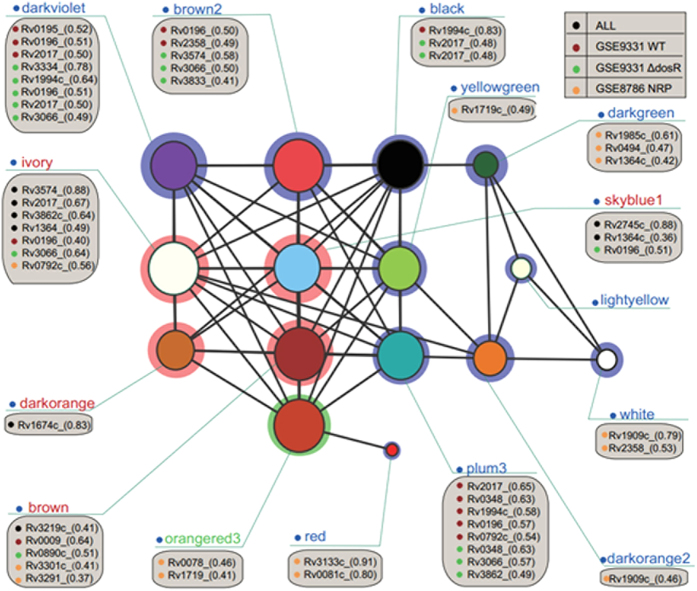
Significantly up-regulated gene modules. Up-regulated gene modules were screened out from the overall network (Fig. 3) and formed a subnetwork. Different modules are shown in different colours and shadowed by three colours: pink shadowing indicates the module was up-regulated in both datasets, blue indicates the module was unique to the dataset it belonged to, and green indicates the module was up-regulated in GSE8786 but down-regulated in GSE9331. By choosing TFs that were highly correlated with the modules and calculating the correlation between their expression profile and each gene module, we could screen them out. The screened TFs for each module are listed in the grey boxes.

**Figure 6:**
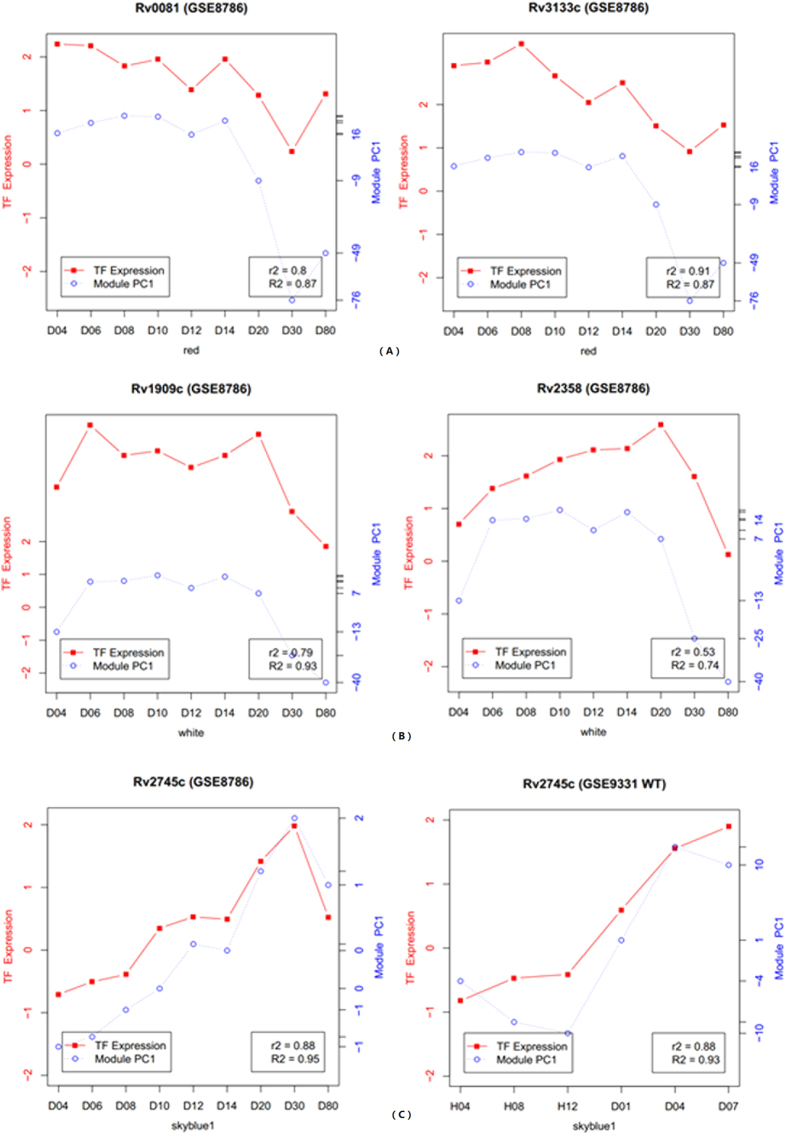
Predict the potential transcription factors (TFs) of gene modules. The abscissa represents the time. Left ordinate indicate a TF’s expression value, which is marked with red points. And the first principal component of module is indicated on the right ordinate and marked with blue points. R2 is the Pearson’s correlation coefficient between TF and the first principal component in the co-expression network, and R2 is their correlation in a particular dataset. (**A**) Correlation schematics of a potential TF (*Rv0081 Rv3133c*) and the first principal component of the red module in GSE8786. (**B**) Correlation schematics of a potential TF (*furA* (*Rv1909c*) *Rv2358*) and the first principal component of the white module in GSE8786. (**C**) Correlation schematics of the TF (*Rv2745c*) and the first principal component of theskyblue1 module in GSE8786 and GSE9331.

To investigate the regulatory mechanisms of these modules in greater detail, we performed a synthesis of the relativity between the first principal component (PC1) of some important modules and the TFs. Based on the GSE9331 and GSE8786 time course analyses, we used the modules related to hypoxia to build subnetworks (Fig. 5). The aim of creating sub network about hypoxia was to show one network’s application, which might bring convenience to solve specific issue, such as hypoxia conditions. The hypoxic sub-network could give us a figure, which showed relationship between hypoxic modules. Meanwhile, we plotted the relationship between TFs and the PC1 of modules in the two datasets in time-course order (Fig. 6). In Fig. 6, we selected TFs in the particular dataset that might potentially regulate the expression of genes in the module.

The results show that MTB responses to the hypoxia models had shared values in some modules and different values in other modules, which provided clues to the stress response mechanism of MTB. For example, when the red and white modules were considered for the role of *DosR* in the Wayne model, their expressive tendencies were very similar. The white module showed analogous expression in the Wayne environment (oxygen concentration gradually changes). Interestingly, the expressions of three modules (dark green, yellow-green, and orange red3) increased with time, indicating that these modules may play roles in different stages during dormancy, implying that the dormancy mechanism of MTB is complex and precise.

### Experimental validation of modules and correlations between potential TFs and their potential targets

#### Experimental validation of modules under hypoxia condition

To verify the modules we predicted, real-time RT-PCR was performed to detect the expression change in the non-replicating persistent NRP2 stage using the H37Rv strain under hypoxia condition. We selected 40 candidate genes in three modules (red, white, and dark violet). We successfully got positive results of 38 genes (40 candidate genes). The selection criteria was that because DosR had been deeply studied, our main purpose of red module was to validate our method is effective. We only selected 4 genes reported. But in white and dark violet module, we selected 22 genes and 12 genes randomly. In two modules, most of genes were not reported that they were associated with hypoxia.

**Figure 7:**
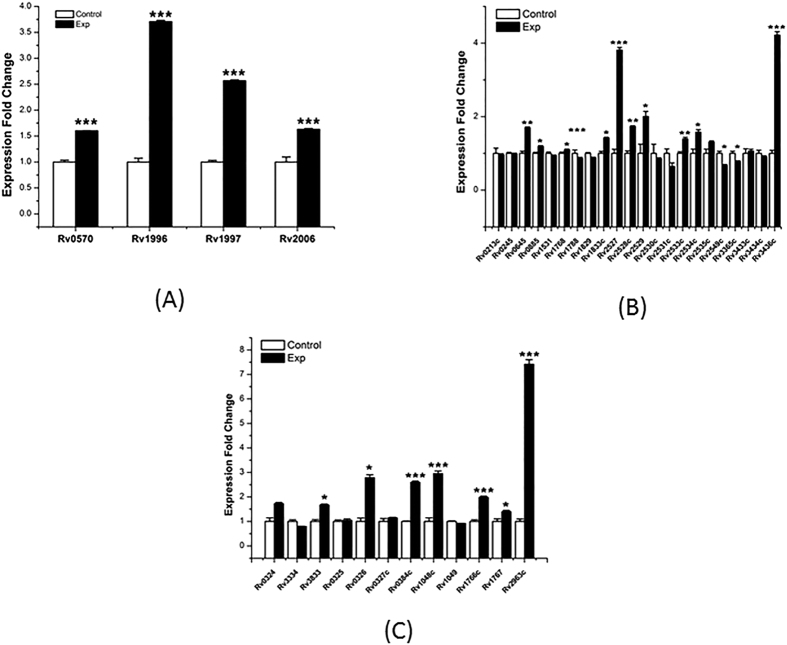
RT-PCR confirmation of expression fold change under hypoxia stress. Expression fold changes of genes in the red (**A**), white (**B**), and dark violet (**C**) modules. RNA samples were extracted at the exponential growth phase (14 days) under normal and hypoxia conditions. The results are shown as average fold changes (hypoxia condition/normal condition). *P < 0.05; **0.01 < P < 0.0; ***P < 0.001.

Among 38 genes, the four selected genes (*nrdZ* (*Rv0570*), *Rv1996, ctpF* (*Rv1997*), and *otsB1* (*Rv2006*)) were significantly up-regulated in the red module(Fig. 7A). In the white module, nearly half the selected genes (*mmaA1* (*Rv0645*)*, Rv0885, PE_PGBS31* (*Rv1768*)*, Rv1833c, Rv2527, mrr* (*Rv2528c*)*, Rv2529, nusB* (*Rv2533c*)*, Rv2534c,* and *Rv3435c*) were significantly up-regulated and three (*Rv1788* (*PE18*)*, Rv2549c,* and *Rv3365c*) were significantly down-regulated (Fig. 7B). In the dark violet module, the majority of the selected genes were significantly up-regulated (*Rv3833, Rv0326, Rv0384c* (*clpB*)*, Rv1048c, Rv1766c, Rv1767,* and *Rv2963c*) (Fig. 7C). The results of anaerobic stimulated experiment indicated that the overwhelming majority genes in the modules we predict were positively related to hypoxia environment.

Although some genes have been reported in these modules (*nrdZ* (*Rv0570*)*, Rv1996, Rv1977, otsB1* (*Rv2006*)*, Rv0885, vapC17* (*Rv2527*)*, clpB* (*Rv0384c*)*, Rv1048c*, and *Rv1766*), there are also some new genes related to hypoxia(*mmaA1* (*Rv0645*)*, Rv0885, PE_PGBS31* (*Rv1768*)*, Rv1833c, Rv2527, mrr* (*Rv2528c*)*, Rv2529, nusB* (*Rv2533c*)*, Rv2534c*, and *Rv3435c*). These genes remains to be studied further.

#### Experimental validation of correlations between TFs and their targets

**Figure 8:**
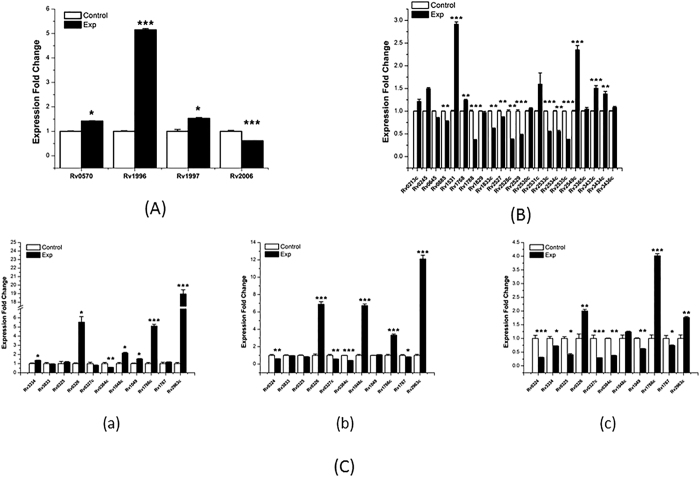
RT-PCR confirmation of expression fold changes related to up-regulated transcription factors under hypoxia stress. (**A**) Overexpressing *Rv0081* up-regulated the expression levels of genes in the red module. (**B**) Impact of overexpressing *furA* (*Rv1909c*) on the expression levels of genes in the white module. (**C**) Up-regulated transcription factors *Rv0324* (i), *Rv3334* (ii) and *Rv3833* (iii) changed the expression levels of genes in the dark violet module. RNA samples were extracted at exponential growth phase (14 days) under normal and hypoxia conditions. The results are shown as average fold change (upregulated plasmid/empty plasmid as control). *P < 0.05; **0.01 < P < 0.05; ***P < 0.001.

Several TFs (*Rv0081, furA* (*Rv1909c*)*, and Rv0324, Rv3334* and *Rv3833*) were present in the three selected modules (red, white, and dark violet). To investigate if these TFs regulate the expression of genes in these modules, we overexpressed the five TFs in H37Rv strain under both hypoxia and normal cultivation conditions and detected the expression level of these genes in the corresponding modules. During the NRP2 stage, overexpression of *Rv0081* up-regulated the expression levels of all the genes in module red (*nrdZ* (*Rv0570*)*, Rv1996, ctpF* (*Rv1997*), and *otsB1* (*Rv2006*)) (Fig. 8A). In the white module, overexpression of *furA* (*Rv1909c*) significantly up-regulated the expression levels of some of the genes (*Rv1531, PE_PGBS31* (*Rv1768*)*, Rv2549c, Rv3433c,* and *Rv3434c*), and significantly down-regulated most of the other genes in this module (*Rv0885, Rv1788* (*PE18*)*, Rv1833c, Rv2527, mrr* (*Rv2528c*)*, Rv2529, Rv2533c* (*nusB*)*, Rv2534c,* and *Rv2535c*) (Fig. 8B). The dark violet module contained three TFs (*Rv0324, Rv3334,* and *Rv3833*). Overexpression of *Rv0324* up-regulated the expression of *Rv3334, Rv0326, Rv1048c, Rv1049, Rv1766c,* and *Rv2963c*, and down-regulated *Rv0384c* (*clpB*) (Fig. 8Ca). Overexpression of *Rv3334* up-regulated the expression of *Rv0326, Rv1048c, Rv1766c* and *Rv2963c*, and down-regulated the expression of *Rv0324, Rv0327c, Rv0384c* (*clpB*), and *Rv1767* (Fig. 8Cc). Overexpression of *Rv3833* significantly down-regulated most of the genes in this module (*Rv0324, Rv3334, Rv0325, Rv0327, Rv0384, Rv1049,* and *Rv1767*), and significantly up-regulated three genes (*Rv0326, Rv1766c,* and *Rv2963c*).

**Figure 9:**
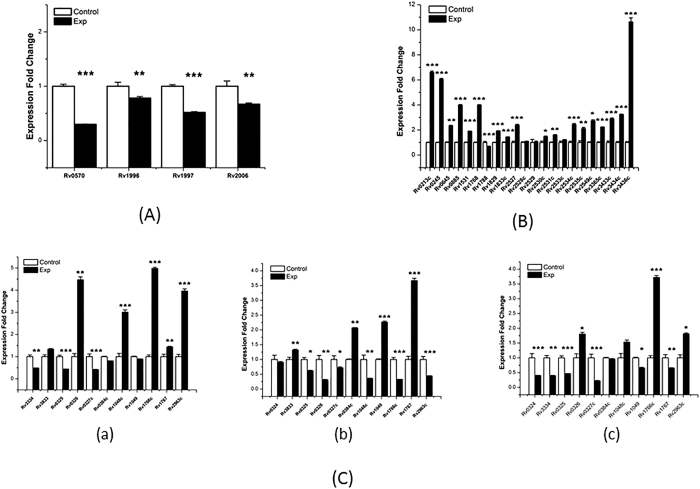
RT-PCR confirmation of expression fold changes related to up-regulated transcription factors under normal conditions. (**A**) Overexpressing *Rv0081* down-regulated the expression levels of genes in the red module. (**B**) Impact of overexpressing *furA* (*Rv1909c*) on the expression levels of genes in the white module. (**C**) Up-regulating transcription factors *Rv0324* (i), *Rv3334* (ii), and *Rv3833* (iii), changed the expression levels of genes in the dark violet module. RNA samples were extracted at exponential growth phase (14 days) under normal and hypoxia conditions. The results are shown as average fold change (upregulated plasmid/empty plasmid as control). *P < 0.05; **0.01 < P < 0.05; ***P < 0.001.

During normal cultivation, overexpression of *Rv0081* significantly down-regulated the expression level of all the genes in the red module (*nrdZ* (*Rv0570*)*, Rv1996, ctpF* (*Rv1997*), and *otsB1* (*Rv2006*)) (Fig. 9A). In the white module, overexpression of *furA* (*Rv1909c*) significantly up-regulated the expression level of most genes (*Rv0213c, Rv0245, mmaA1* (*Rv0645*)*, Rv0885, Rv1531, PE_PGBS31* (*Rv1768*)*, Rv1829, Rv1833c, Rv2527, Rv2530c, Rv2531c, Rv2534c, Rv2535c, Rv2549c, Rv3365c, Rv3433c, Rv3434c,* and *glmS* (*Rv3436c*)), and significantly down-regulated *Rv1788* (*PE18*) (Fig. 9B). The dark violet module contains three TFs (*Rv0324, Rv3334,* and *Rv3833*). Overexpression of *Rv0324* up-regulated the expression of *Rv0326, Rv1048c, Rv1766c, Rv1767,* and *Rv2963c*, and down-regulated *Rv3334, Rv0325,* and *Rv0327c* (Fig. 9Ca). Overexpression of Rv3334 up-regulated the expression of four genes in this module (*Rv3833, Rv0384c* (*clpB*)*, Rv1049,* and *Rv1767*) and down-regulated six genes (*Rv0325, Rv0326, Rv03827c, Rv1048c, Rv1766c,* and *Rv2963c*) (Fig. 9Cb). Overexpression of *Rv3833* significantly down-regulated most genes in this module (*Rv0324, Rv3334, Rv0325, Rv0327, Rv1049,* and *Rv1767*) and significantly up-regulated three genes (*Rv0326, Rv1766c,* and *Rv2963c*) (Fig. 9Cc).

## Discussion

Correlation networks are increasingly being used in bioinformatics applications. For example, weighted gene co-expression network analysis is a systems biology method for describing the correlation patterns among genes across microarray samples. Weighted correlation network analysis (WGCNA) can be used for finding clusters (modules) of highly correlated genes, for summarizing such clusters using the module eigengene or an intramodular hub gene, for relating modules to one another and to external sample traits (using eigengene network methodology), and for calculating module membership measures. We think this method is well-established.

Using the WGCNA algorithm, we identified gene modules in which the genes showed similar expression trends and were governed by common TFs with a genome-wide perspective. Consequently, these modules can serve as credible data for transcriptional analyses of MTB and aid research into the functions of the MTB genes.

The major data sources for this study included GSE1642^13^ (437 microarrays), exptsetno_4615 (814 microarrays, unpublished), a single-channel biochip concerning TF overexpression (100 microarrays, published by Galagan *et al*.^3^), and some hypoxia and NO stress biochips. We obtained satisfactory co-expression data from each of them individually; however, when we merged them, similar co-expression data were difficult to generate because of the fusion of several modules. The merger of all biochips inevitably reduced the number of genes, which was less than 3000 and did not cover the entire MTB genome.

For a single dataset with various experimental conditions (e.g., GSE1642 and exptsetno_4615), genes in different pathways and with regulatory mechanisms showed different expression trends; thus, most of these genes were separated and assigned to different modules. Because differences within datasets had more influence on the gene modules than differences within the biochips, the merger of some datasets resulted in some important modules being missed. Furthermore, if we had simplified the experimental conditions instead of diversifying them, then only modules associated with specific conditions would be identified. Modules have been used successfully in previous studies^14^,^15^,^16^.

When merged bigger dataset, we found the number of genes was less than 3000, which could not cover MTB genome. We wanted to get the co-expression modules on numerous conditions, but not a condition. So we collected microarray data from a variety of experimental conditions to build small dataset by ourselves. therefore, we collected microarray data from a variety of experimental conditions and analysed the gene expression profiles differently. We chose biochips from a number of research programs (the biochips from each program could not be too large) and three datasets related to hypoxia, *DosR* mutations, and intracellular infection models in mice. By merging the data from these biochips, we obtained a gene expression matrix with 303 rows (biochips) and 3411 columns (genes). After filtering the biochip data, we constructed a co-expression network based on the new gene expression matrix. To improve the specificity and accuracy of the co-operation analysis results, we plan to investigate how the difficulties concerning modular fusion in larger datasets can be overcome.

Although *DosR* is the best studied TF in MTB^17^,^18^,^19^, it is still unclear how MTB reacts and adapts to low-oxygen stress. In general, *DosR* is necessary for MTB’s reaction to and survival in a hypoxic environment; however, in the Rustad *et al*. study (GSE9331)^9^, Rustad *et al*. proposed that the *DosR* regulon was the only option for MTB in response to a low-oxygen environment *in vitro*, but that *DosR* was not the initial or main method.

But in the conclusion of GSE8786, it was clear that the growth defect of *DosR* mutants started after MTB entered the NRP2 (non-replicating persistence 2) stage. These observations also suggested that *DosR* plays a major role in regulating MTB entry into anaerobic dormancy. Interestingly, most genes in the *DosR* regulon maintained a high level of expression in NRP2 and the expression reduced only after 30 days. At last, they concluded that the *DosR* regulon was not necessary for dormancy, but influenced the process of entering the dormancy state.

Based on the results of expression responses at the module level in the two hypoxia models, we infer that there are different master regulator TFs involved in two hypoxia models. The genes in these modules, which have similar expression profiles, can be considered to be regulated in a general way. Besides, Wayne model condition and constant hypoxia condition could activate different master TFs and then lead to specific expression profiles of many modules in two models, which remain to be furtherly explored.

*DosR* is the regulatory protein of a 3-component system. In hypoxia, self-phosphorylation of *DosT* and *DosS*, the receptor sensor histidine kinase, could promote *DosR* transcriptional activity. The difference between the constant hypoxia model and the Wayne growth model was neither the oxygen concentration (the oxygen concentration of the Wayne model at the beginning was only 0.2% higher than the constant hypoxia condition) nor the length of time, but the rate of oxygen concentration variation. This finding demonstrated that *DosT* probably can respond to changes in hypoxia, but not to hypoxia itself. In GSE9331, *DosR* increased significantly in the first four hours because of the sudden decrease in the oxygen concentration, and later returned to its normal level. In GSE8786, where the oxygen concentration continued to drop, *DosR* expression was kept at a high level; however, when MTB entered into the NRP2 stage the bacteria were almost completely inactive (the turbidity showed no further increase). Thus, the lack of oxygen consumption either led to a stable oxygen concentration or the concentration was too low to induce a reaction from the two-component system, which lost regulatory activity causing the *DosR* regulon to be down-regulated. We concluded that the *DosR* family was sensitive to the rate of oxygen concentration variation, rather than to the hypoxic condition. We would use experiment to prove our conclusion.

From the time-course analysis at the module level, we identified important modules and predict the functions of the gene in these modules. For simplicity, we selected modules that were significantly up-regulated in GSE8786; for example, the orangered3 module that contained 15 genes, including eight genes encoding a ribosomal protein and two genes encoding parts of the RNA polymerase (*rpoB* and *rpoC*).

We plan to further filter the regulatory factors and normal coding genes that were highly related to this module in GSE8786 to help reveal the vital gene functions and regulation mechanisms of dormancy in MTB.

In the validation experiment we predict that three modules (red, white, and dark violet) are related to latency and showed that the genes in these modules were induced under anaerobic conditions. Accordingly, the predictions of the latent module are correct.

Under anaerobic or normal conditions, overexpression of TFs can have a great influence on the expression of genes in these modules, indicating that many of the genes in these modules are targets of the TFs, indicating that the TFs regulate genes in the module.

Our results also showed that the regulation patterns of the TFs were different in different cultivation conditions. Overexpression of different TFs led to different results in the different modules; for example, in the dark violet module, overexpression of *Rv3833* significantly down-regulated most of the genes under anaerobic and normal conditions while overexpression of *Rv0324* and *Rv3334* in the same module had the opposite effect. The different expression profiles between the TF regulations in the same module may indicate that different co-factors participate in the process of regulating downstream genes in different cultivation environment. Further experiments are required to confirm this point. TFs within the same module may restrict each other’s expression; for example, the overexpression of *Rv3833* in the dark violet module suppressed the expression of *Rv0324* and *Rv3334* under the two aerobic stresses.

Our results indicate that one particular TF in a module may only partially regulate the genes in that module. The expression profiles of the genes within a module are affected by multiple factors, including multiple TFs and the other unknown factors. Thus, each module consisted of various TFs working together under different regulatory models.

Our main purpose of validation is to verify our prediction. If we verify our prediction successfully, the co-expression network is available and reliable.

In this paper, we created co-expression network in MTB and performed some applications in module level. Meanwhile, we predicted potential transcriptional regulatory factors and identified new hypoxic modules. But the issue about that how these different TFs ultimately govern other genes in detail that still need to address. We would do our best to clarify the mechanism in the future.

## Methods

### Data sources

#### Microarrays

The microarray datasets were selected using the following criteria. (i) The microarray must be for the MTB H37Rv strain and be a double-channel microarray. (ii) The microarray datasets must meet our quality requirements, which we assessed using the WGCNA^10^,^11^ and array Quality packages. (iii) There is as high as possible diversity of the experimental conditions. (iv) The number of each condition of the microarrays should be balanced. The microarray gene expression profiles used in this study were: GSE5977, GSE6750, GSE7962, GSE8664, GSE8786, GSE8689, GSE8732, GSE8830, GSE9776, GSE13978, GSE13998, GSE15976, GSE17640, GSE30299, GSE46212, GSE6209, and GSE11096^16^,^20^,^21^,^22^,^23^,^24^,^25^,^26^,^27^,^28^,^29^,^30^,^31^,^32^,^33^.

#### Annotation databases

We used the Kyoto Encyclopedia of Genes and Genomes (KEGG) database ( www.genome.jp/kegg/), the Gene Ontology (GO) database ( www.geneontology.org/).

#### Testing dataset

TF target gene set from the MTB regulatory network constructed by Sanz *et al*.^5^ for the enrichment analysis of gene modules. The dataset contains 3411 genes, which do not cover genome-wide genes. Compared with paper’s data set, there are 15 genes missing in *DevR* set, and also 26 genes missing in *KstR* set.

### Processing of the microarray data

The R Bioconductor limma package was used to preprocess the raw microarray data as follows. (i) The probe ID was replaced by the gene number in the raw microarray data, and then the original data were read. (ii) The normalize Within Arrays function with loess (chip data without point information) or Print-tip loess (chip data with point information) was used to normalise the data. (iii) The mean and standard deviation (SD) of background values were calculated, and if a probe value was ≤ the background mean value +1*SD, the probe was marked as NA (low signal). (iv) The data were merged to create a new dataset by gene names.

### Construction of the co-expression network

We accumulated and preprocessed the raw data from biochips that had used strain H37Rv to construct the co-expression network. The construction and analysis of the co-expression network were based on WGCNA, which is a typical algorithm. In the co-expression network, the nodes represent the gene module and the lines indicate the relevance of the co-expression modules. A module refers to a set of genes with similar expression trends in different samples. In the WGCNA algorithm, the elements in the co-expression matrix are defined as the weighted value of the correlation coefficient.

The network was built based on the connectivity between nodes. The selection for the weighted value is that network could satisfies the scale-free law^34^. *i* means node links (in other words, node degree *i*). *p*(*i*) means that the probability that a node has exactly *i* links (in other words, degree *i*) follows a power law distribution, namely *p*(*i*) ~ *i*^−*n*^ where *i* is the node connectivity and *p* is the probability. In practical applications, the network is made to approximate the scale-free law by selecting weighting coefficients. The log(*i*) and log(*p*(*i*)) values should have a negative correlation (>0.8).

We used the WGCNA package to construct the co-expression network as follows:

(a) Define gene co-expression similarity: Calculate the similarity between any two genes using Pearson’s correlation coefficient (*S*_*ij*_ = |cor(*i*, *j*)|, the correlation coefficient of gene *i* and gene *j*), which then forms the correlation matrix (*S* = [*S*_*ij*_]).

(b) Define the exponential weighted value *β*: For any gene pair (*i* and *j*), apply the exponential adjacency function in the WGCNA algorithm to measure their relation index, namely, the exponential weighted *β* square of the correlation coefficient (*a*_*ij*_ = *power*(*S*_*ij*_, *β*) = |*S*_*ij*_|^*β*^). Exponential weighted β is the power of the correlation coefficient. We selected *β* = 5 after the analysis (fit value R^^2^ to approximately 0.9) shown in Fig. 1

(c) Define a measure of node dissimilarity: After determining the adjacency function parameter *β*, the correlation matrix *S* = [*S*_*ij*_] is switched into the adjacency matrix *A* = [*a*_*ij*_] and converted into the topological overlap matrix Ω = [*ω*_*ij*_]. *k*_*i*_ or *k*_*j*_ indicate the sum of one node’s adjacency coefficients. The node is a gene (*i* or *j*).


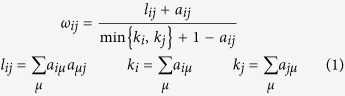


(d) Build hierarchical clustering tree to identify gene modules: The hierarchical clustering tree built using the dissimilarity coefficient 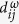 (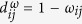), and the different branches represent the gene modules.

(e) Construct the co-expression network:

We got 78 gene modules by the function cutreeDynamic in WGCNA package. The soft thresholding power was 5. The relatively minimum module size is 10, and we chose a medium sensitivity (deepSplit = 2) to splits cluster. By the Pearson correlation coefficient between modules, we constructed the network. When the absolute value of correlation was more than 0.45, we would link two modules. The co-expression network was constructed.

### Gene module enrichment and significant analysis of co-expression gene module

Fisher’s exact test was used for the gene set enrichment analysis. The analysis tended to cluster genes with similarities into one module of the co-expression network. To determine the reliability of the network module results, we formed a simulation dataset by random permutations, and then calculated the probability of the GO terms, KEGG pathways, and TFs in another regulation network enriched in our gene modules.

Using the KEGG enrichment analysis as an example, the progress of the significant co-expression network analysis is shown in detail below:

(a) Calculate the enriched KEGG pathways in each gene module (*P* value < 10^−5^) as *n*.

(b) Take all the genes in the dataset as an overall sample and form a simulated module set according to the number of genes in each module. Repeat the calculation in (a) with a lower threshold (*P* value < 10^−2^) and save as *n*_*i*_.

(c) Repeat step (b) 1000 times to get a set of numbers of significantly enriched KEGG pathways.

(d) Calculate the *Z* value (Table 2) as


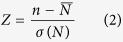


n: In a repeat, the sum of numbers of all KEGG enrichment pathway

N: if repeat 1000 times, N = (n1, n2 …n1000).

### Time-course analysis by the NetReSFun algorithm at the module level

We modified NetReSFun^2^,^4^ to identify the module related to sequential response of MTB under hypoxic pressure. In general, NetReSFun estimates where there is a change of expression in the interval [*τ* − 1, *τ*]. The scaled covariance between gene expression profiles could show the change directly.

Firstly, suppose there is a function step from 0 to 1 at time *τ* as follows:


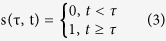


Secondly, with the expression profile *x*_*i*_(*t*) of gene *i* and the function s(τ, t), the scaled covariance *cov*_*i*_(*t*) is:


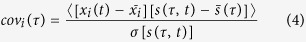


(The average of all times is represented by the short string in above function and the average of the sum of the product is represented by the curly brace in above function).

Thirdly, to ensure only the change of gene expression influences the final results, define *Z* as:


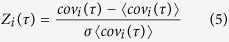


(where *Z*_*i*_(*τ*) represents the transcriptional response of gene *i* during [*τ* − 1, *τ*]). Similarly, *Z* could represents the response of gene module *I* at time *τ* as:


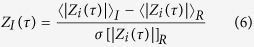


(The subscripts *I* and *R* represent the mean value of the genes these modules contains and the mean value of the same number of randomly selected gene, respectively).

So, when *Z*_*I*_(*τ*) ≥ 1.65 (log10 can be transformed to 0.22), it could be considered that the gene module has a significant change in expression level during *τ* − 1, *τ*.

Fourthly, to determine the direction of the change, the bias *R* of positive and negative covariance of genes within the module is calculated as:


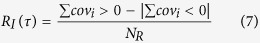


(where *N* represents the number of genes a module contains).

At last, the *Z* value is calculated to measure the significance of *R* as:


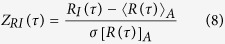


(where subscript *A* represents all the other modules, and the curly brace in above function represents the average *R* of all the other gene modules).

In Original NetReSFuns, they only could identify whether expression of genes are abnormal, but we could not ensure that gene was up-regulated or down-regulated. We remove absolute value of *Z*_*RI*_(*τ*). So, *Z*_*RI*_(*τ*) > 0 indicates the module was up-regulated during [*τ* − 1, *τ*], otherwise it was down-regulated.

### Screening potential TFs of gene modules

TFs may control the different gene modules under different conditions. To analyse a particular gene module, we selected all the TFs with a correlation value > 0.35 with this module. We then calculated the correlation between each TF and the first principal component of the module; high correlation suggested a high possibility that the module is controlled by the particular TF. Finally, we identified the possible TFs associated with a particular gene module and then considered the expression levels of these TFs.

### Construction of an overexpression plasmid

To create pMV261-Rv0081, pMV261-Rv1909c, pMV261-Rv0324, pMV261-Rv3334, and pMV261-Rv3833 constructs, the sequences of the *Rv0081, furA* (*Rv1909c*)*, Rv0324, Rv3334,* and *Rv3833* genes were amplified by polymerase chain reaction (PCR) from MTB H37Rv genomic DNA using primers presented in follow Table 5. The genes were then cloned into the pMV261 plasmid (which is a gift from Prof Jun Liu, University of Toronto, Canada) between the *BamHI* and *EcoRI* restriction sites using standard methods. Primers used to amplify the MTB genes was shown in Table 5.

### *Escherichia coli* and MTB strains, media, and growth conditions

MTB H37Rv were growth in liquid Middlebrook 7H9 medium and cultured in test tubes at 37 °C without extra oxygen until the exponential phase of growth reached standard conditions. The cultures were then subjected to an anaerobiosis stages as described by Wayne and Hayes^35^. In the hypoxia model, 2/3 volume of test tubes were added with liquid medium before sealed. As a control, the cultures were grown under standard conditions on 1/3 volume of test tubes with liquid medium without sealing them. The strained solutions (turbidity at 10 mg mL^−1^) from each test tube were then added to test tubes at 1/100 volume. The H37Rv bacteria grown under standard and hypoxic conditions were used for RNA isolation. In the Wayne model experiments, the cultures kept 15 days in an anoxic environment. On 15th day, RNA were extracted.

*E. coli* strain DH5α was grown on Luria broth at 37 °C. Then, 50 μg mL^−1^ kanamycin was added for selection.

### RNA extraction and reverse-transcription (RT)-PCR validation

The selected strains were centrifuged at 4,500 × g for 5 min at room temperature, and frozen on dry ice. The frozen cell pellets were suspended in 1 mL TRIzol reagent (CW Bio). RNA extraction was performed as previously described^36^. Genomic DNA was removed using a PrimerScript™ RT reagent kit (Takara).

Reverse transcription was performed with random primers, and quantitative PCR was performed with SYBR green mix (CW Bio).

We chose 40 candidate genes for RT-PCR validation. Because *DosR* has been deeply studied, our main purpose of red module is to validate our method is effective. We only selected 6 reported genes in red module. But in white and dark violet module, we randomly selected 22 genes and 12 genes separately for validation.

The primer sequences of candidate genes used for the RT-PCR are available upon request.

## Additional Information

**How to cite this article**: Jiang, J. *et al*. Construction and application of a co-expression network in *Mycobacterium tuberculosis*. *Sci. Rep.* **6**, 28422; doi: 10.1038/srep28422 (2016).

## Supplementary Material

Supplementary Information

Supplementary Information

## Figures and Tables

**Table 1:** Correlation analysis between the gene modules and the target gene sets of the regulation factors, *DosR* and *KstR*.

	TFs	Intersection number	Number of module genes	Number of testing gene set	*P*-value
1^st^	*DosR*	40	88	45	1.04E-62
	*KstR*	19	28	53	7.24E-30
2^nd^	*DosR*	31	49	45	5.11E-52
	*KstR*	15	25	53	2.52E-22

The target gene set of the regulation factor come from the intersection of the target gene sets and the 3411 genes in the dataset. The second network is the co-expression network with the microarray data from 233 biochips after ruling out the microarray data on the hypoxia, intracellular, infected mouse model, and dosR mutation conditions. The first network is the co-expression network using all 303 microarray biochips.

**Table 2:** Differences between the enrichment of specific gene sets by gene modules and random modules.

	GO	KEGG	TFs target
The number of genes enriched by experiment gene modules	13	6	26
The average number of genes enriched by random gene modules	1.96	1.49	1.44
The variances of the number of genes enriched by random gene modules.	1.46	1.46	1.23
Z-Value	7.56	3.08	19.94
p-Value	2.02E-14	0.001	<1.00E-16

The first line is the number of genes in the GO, KEGG, and TF target gene sets enriched by 78 gene modules. The second line is the average number of genes enriched by 78 random modules in 1000 replications. The third line is the variance of genes enriched by 78 random modules in 1000 replications. The fourth and fifth lines are the *Z* value and corresponding P value of the comparison between experimental data and random data. Considering the low intensity of genes enriched by the random modules, we used P < 0.01 as the significant threshold, while for the experimental gene modules we used P < 10^−5^ as the significant threshold. Fisher’s exact test was used for the enrichment analysis calculation.

**Table 3:** Target gene sets of regulation factors enriched by the gene modules.

Gene set	Gene Module	p Value	Gene Set	Gene Module	p Value
*Rv0260c*	mediumorchid	1.88E-08	*Rv2034*	plum3	2.77E-07
*Rv0348*	magenta	3.94E-10	(*sigC*) *Rv2069*	skyblue2	3.65E-14
	orangered3	3.19E-07	(*furB*) *Rv2359*	blue2	8.18E-11
	red	9.37E-10		skyblue2	1.83E-19
*hspR* (*Rv0353*)	plum3	8.68E-07	(*ideR*) *Rv2711*	lightcyan1	6.62E-36
*regX3* (*Rv0491*)	skyblue2	1.85E-10		skyblue2	2.13E-12
*Rv0494*	magenta	2.20E-12	(*virS*) *Rv3082c*	green	8.80E-09
(*phoP*) *Rv0757*	navajowhite2	1.92E-07	(*devR*) *Rv3133c*	red	1.23E-54
	turquoise	9.72E-08	(*sigH*) *Rv3223c*	darkorange	3.89E-16
*Rv0818*	mediumorchid	1.75E-07	(*sigF*) *Rv3286c*	firebrick4	2.61E-07
(*mprA*) *Rv0981*	magenta	1.41E-14	(*whiB3*) *Rv3416*	plum3	1.89E-09
	red	3.33E-15	*Rv3557c*	mediumorchid	3.75E-08
(*sigE*) *Rv1221*	skyblue2	7.66E-07	(*kstR*) *Rv3574*	ivory	2.86E-33
*Rv1359*	mediumorchid	3.08E-06	*Rv3849*	navajowhite2	2.18E-07
(*argR*) *Rv1657*	firebrick4	4.25E-10	(*sigM*) *Rv3911*	grey60	6.82E-07
*Rv1931c*	mediumorchid	1.59E-06		navajowhite2	2.41E-08
(*mce3R*) *Rv1963c*	darkgreen	7.38E-08			

Fisher’s exact test is used for the enrichment analysis calculation. The significant threshold was set as P < 10^−5^.

**Table 4:** Gene module enrichment in the KEGG and GO datasets.

Database	geneset	annotation	module	*p*-value
GO	GO:0001101	Response to acid	green	5.19E-07
	GO:0001666	Response to hypoxia	red	2.09E-11
	GO:0006412	Translation	salmon4	9.54E-15
			orangered3	1.86E-09
			blue	3.65E-05
			blue2	9.68E-04
	GO:0010033	Response to organic substance	mediumorchid	3.90E-04
	GO:0010106	Cellular response to iron ion starvation	lightcyan1	2.47E-06
	GO:0040007	Growth	orangered3	2.13E-05
			salmon4	3.09E-04
	GO:0044117	Growth of symbiont in host	magenta	7.19E-06
			lavenderblush3	8.41E-04
	GO:0052572	Response to host immune response	lightcyan1	9.96E-04
	GO:0071500	Cellular response to nitrosative stress	red	1.24E-20
	GO:Unannoted		grey60	2.71E-06
			darkgreen	9.75E-04
	GO:0005622	Intracellular	salmon4	2.95E-16
			orangered3	2.99E-09
			blue	3.27E-04
	GO:0005840	Ribosome	salmon4	1.66E-15
			orangered3	6.42E-10
			blue	4.39E-05
			blue2	6.30E-04
	GO:0005886	Plasma membrane	salmon4	1.55E-04
	GO:0003735	Structural constituent of ribosome	salmon4	1.66E-15
			orangered3	6.42E-10
			blue	4.39E-05
			blue2	6.30E-04
	GO:0005515	Protein binding	blue	8.32E-04
	GO:0008137	NADH dehydrogenase activity	magenta	6.40E-14
	GO:0046933	Proton-transporting ATP synthase activity, rotational mechanism	plum2	1.30E-07
	GO:0046961	Proton-transporting ATP synthase activity, rotational mechanism	plum2	1.30E-07
KEGG	mtu00190	Oxidative phosphorylation	magenta	3.60E-07
			plum2	1.94E-04
	mtu00281	Geraniol degradation	lavenderblush3	2.42E-07
	mtu00330	Arginine and proline metabolism	firebrick4	1.03E-07
	mtu00362	Benzoate degradation	ivory	4.30E-04
	mtu00400	Phenylalanine, tyrosine and tryptophan biosynthesis	darkolivegreen4	1.54E-05
	mtu00621	Dioxin degradation	ivory	6.40E-04
	mtu00623	Toluene degradation	darkmagenta	2.64E-04
	mtu00626	Naphthalene degradation	lavenderblush3	1.80E-06
	mtu00670	One carbon pool by folate	midnightblue	5.98E-04
	mtu00760	Nicotinate and nicotinamide metabolism	darkturquoise	5.60E-04
	mtu01053	Biosynthesis of siderophore group nonribosomal peptides	lightcyan1	4.79E-12
	mtu03010	Ribosome	salmon4	3.94E-21
			orangered3	1.12E-09
			blue	9.17E-05
			blue2	7.88E-04
	Unannotated		grey60	5.57E-05

Fisher’s exact test was used for the enrichment analysis calculation (P < 10^−3^).

**Table 5:** Primers used to amplify the MTB genes.

Forward primer	Sequence (5′ –> 3′)	Reverse primer	Sequence (5′ –> 3′)
pMV261-Rv0081-F	CGCGGATCCGTGGAGTCCGAACCGC	pMV261-Rv0081-R	CCGGAATTCGTGGCCGAGCCGCCGG
pMV261-Rv1909c-F	CGCGGATC GTGTCCTCTATACCGG	pMV261-Rv1909c-R	CCGGAATTCGGATGTGATCGCGAAGTG
pMV261-Rv0324-F	CGCGGATCCATGGCTGGACAGTCCG	pMV261-Rv0324-R	CCGGAATTATCCCCATGCCCGACCG
pMV261-Rv3334-F	CGCGGATCAAGATCAGCGAGGTAGC	pMV261-Rv3334-R	CCGGAATTCGGCGTGAATGTCGCTGA
pMV261-Rv3833-F	CGCGGATCTCGGAAAACAGCCACC	pMV261-Rv3833-R	CCGGAATTCGGCGATCGCGAGCGCG
