# Corrigendum: Construction and application of a co-expression network in *Mycobacterium tuberculosis*

**DOI:** 10.1038/srep40563

**Published:** 2017-01-12

**Authors:** Jun Jiang, Xian Sun, Wei Wu, Li Li, Hai Wu, Lu Zhang, Guohua Yu, Yao Li

Scientific Reports 6: Article number: 2842210.1038/srep28422; published online: 06 22 2016; updated: 01 12 2017

A supplementary dataset containing 3411 genes and their expression profiles from 303 microarrays available in the public domain, Gene Expression Omnibus (GEO) was omitted from the original version of this Article. This has been corrected in the HTML version of the Article; the PDF version was correct at time of publication.

