# Supplementary material for: Construction and application of a co-expression network in Mycobacterium tuberculosis
Source: Sci Rep. 2016 Jun 22;6:28422. doi: 10.1038/srep28422 (PMC4916473; doi:10.1038/srep28422)
Supplement: Supplementary Information [file srep28422-s1.pdf]

Construction and application of a co-expression network in *Mycobacterium tuberculosis*

Short title: Co-expression network in *M. tuberculosis*

Jun Jiang[§], Xian Sun[§], Wei Wu, Li Li, Hai Wu, Lu Zhang, Guohua Yu*, Yao Li*

State Key Laboratory of Genetic Engineering, Shanghai Engineering Research Center of Industrial Microorganisms, School of Life Sciences, Fudan University, Shanghai 200438, China

[§]These authors contributed equally to this work.

*Corresponding authors: Tel: 86-021-51630559. E-mail addresses: yaoli@fudan.edu.cn (Li) and 10110700066@fudan.edu.cn (Yu)

The gene module motif:

module number	The number of genes	top 20 genes in each module by connection degree	Potential regulatory motif
M1	19	Rv0462，Rv0483，Rv2867c，Rv2340c，Rv1163，Rv1162，Rv0872c，Rv1387，Rv0905，Rv3165c，Rv0041，Rv1803c，Rv1555，Rv2986c，Rv1386，Rv0120c	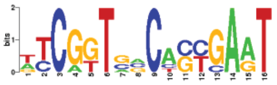
M2	26	Rv3401， Rv3410c， Rv2604c， Rv2605c， Rv3361c，Rv2216，Rv1660，Rv2750，Rv0335c，Rv0530， Rv3375， Rv3899c， Rv3718c， Rv2315c，Rv0488，Rv1958c，Rv1986，Rv3308，Rv1284， Rv1144…	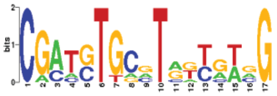
M3	87	Rv3175，Rv0790c，Rv0990c，Rv1050，Rv2642，Rv0792c，Rv0758，Rv2641，Rv3174，Rv0791c，Rv1994c， Rv1992c， Rv3176c， Rv0331， Rv1993c，Rv3178，Rv0768，Rv3760，Rv0687，Rv2618c…	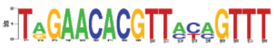
M4	178	Rv0951，Rv0732，Rv0635，Rv0637，Rv1379，Rv2445c，Rv0056，Rv0636，Rv0055，Rv0567，Rv0466，Rv2412，Rv0203，Rv0054，Rv3924c，Rv0053， Rv2441c， Rv3646c， Rv2462c， Rv3137c…	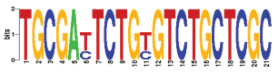
M5	12	Rv2056c， Rv2057c， Rv2058c， Rv2055c， Rv0106，Rv2059，Rv1087，Rv2060，Rv0281，Rv3321c，Rv2727c，Rv1507c	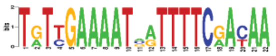
M6	149	Rv0951，Rv0732，Rv0635，Rv0637，Rv1379，Rv2445c，Rv0056，Rv0636，Rv0055，Rv0567，Rv0466，Rv2412，Rv0203，Rv0054，Rv3924c，Rv0053， Rv2441c， Rv3646c， Rv2462c， Rv3137c…	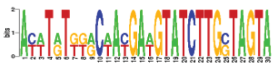
M7	13	Rv1922， Rv3530c， Rv1282c， Rv3531c， Rv2330c， Rv3571， Rv1587c， Rv1588c， Rv1628c，Rv1702c，Rv2100，Rv3529c，Rv1281c	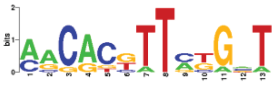
M8	26	Rv0974c， Rv0789c， Rv2666， Rv0975c， Rv0109， Rv0976c， Rv0679c， Rv0972c， Rv2087， Rv2660c， Rv1153c， Rv1575， Rv1329c， Rv1216c， Rv2661c， Rv0680c， Rv3737，Rv2288，Rv0484c，Rv1053c…	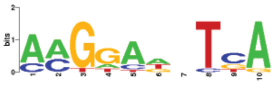
M9	20	Rv1332，Rv0231，Rv3856c，Rv2471，Rv2675c，Rv2676c， Rv2919c， Rv0566c， Rv0583c， Rv1333，Rv1396c，Rv0865，Rv3169，Rv0240，Rv0336，Rv0761c，Rv2051c，Rv0130，Rv0900，Rv2924c	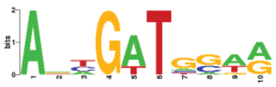

M10	19	Rv3360， Rv0323c， Rv3047c， Rv2510c， Rv0756c， Rv2977c， Rv1112， Rv1926c， Rv2900c，Rv0754，Rv2997，Rv2548，Rv1105，Rv2787，Rv2253，Rv2667，Rv3638，Rv1517	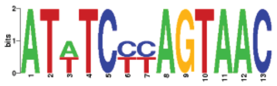
M11	57	Rv3438，Rv3213c，Rv3774，Rv1240，Rv1242，Rv2114，Rv2046，Rv3725，Rv3224，Rv1400c，Rv1175c，Rv0879c，Rv0654，Rv0460，Rv0432，Rv2495c， Rv2457c， Rv2068c， Rv1932， Rv1689…	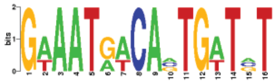
M12	47	Rv0375c， Rv0258c， Rv0374c， Rv0387c， Rv2804c， Rv0104， Rv1572c， Rv0836c， Rv2810c，Rv1551，Rv0797，Rv1490，Rv1961，Rv1873，Rv1675c，Rv3428c，Rv0397，Rv3845，Rv1970，Rv1975…	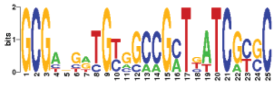
M13	44	Rv1372，Rv2491，Rv0395，Rv2044c，Rv3352c，Rv1966，Rv0648，Rv1371，Rv0842，Rv1965，Rv2319c， Rv0796， Rv0355c， Rv1999c， Rv3181c， Rv3351c， Rv0776c， Rv1268c， Rv2040c，Rv2167c…	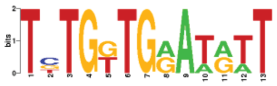
M14	35	Rv2654c， Rv1902c， Rv0458， Rv1553， Rv2907c， Rv2406c， Rv1903， Rv2653c， Rv1904，Rv0376c，Rv1552，Rv0319，Rv0878c，Rv2932，Rv0459，Rv3651，Rv1439c，Rv0655，Rv1554，Rv3894c…	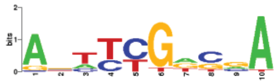
M15	37	Rv2760c，Rv2761c，Rv1851，Rv1947，Rv1610，Rv0899，Rv2759c，Rv1712，Rv1550，Rv1522c，Rv2962c， Rv1876， Rv3033， Rv1563c， Rv2763c， Rv2758c， Rv1505c， Rv3268， Rv1562c，Rv3263…	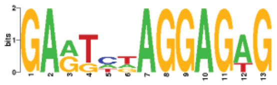
M16	13	Rv2539c， Rv1476， Rv2920c， Rv3509c， Rv2540c， Rv2537c， Rv3788， Rv1248c， Rv3099c，Rv2538c，Rv3389c，Rv1017c，Rv0740	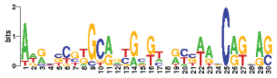
M17	42	Rv0142，Rv1335，Rv3913，Rv0141c，Rv1673c，Rv3463， Rv0816c， Rv1334， Rv1528c， Rv1674c， Rv3222c， Rv0794c， Rv0140， Rv1471， Rv3206c， Rv3054c， Rv2399c， Rv2398c，Rv1336，Rv2397c…	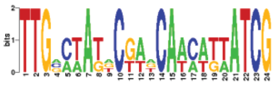
M18	26	Rv1798，Rv0347，Rv1985c，Rv3430c，Rv0671，Rv0658c， Rv0235c， Rv1354c， Rv1077， Rv1501，Rv1847，Rv0329c，Rv1499，Rv0363c，Rv2584c， Rv0132c， Rv1020， Rv3278c， Rv0670，Rv3315c…	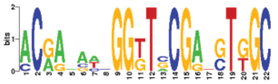

M19	49	Rv1602，Rv1600，Rv0311，Rv0342，Rv3633，Rv3339c， Rv3878， Rv1377c， Rv3584， Rv2096c， Rv3291c， Rv3476c， Rv1765c， Rv2407，Rv2885c，Rv2579，Rv1391，Rv3226c，Rv0505c， Rv2187…	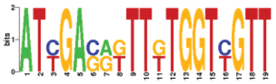
M20	20	Rv0729，Rv1187，Rv3778c，Rv3630，Rv1193，Rv1591，Rv1590，Rv3850，Rv1385，Rv2306c，Rv1208，Rv3851，Rv3917c，Rv3293，Rv2221c，Rv3744， Rv1544， Rv2443， Rv2726c	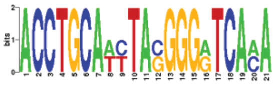
M21	25	Rv1339，Rv1603，Rv0383c，Rv1604，Rv1540，Rv1605， Rv1843c， Rv3607c， Rv0897c， Rv1539，Rv2226，Rv0337c，Rv3255c，Rv1606，Rv1091， Rv1637c， Rv3706c， Rv0293c， Rv2899c， Rv2979c…	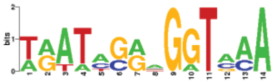
M22	45	Rv2733c，Rv1782，Rv1559，Rv3921c，Rv0734，Rv0554，Rv0155，Rv0009，Rv1783，Rv3200c，Rv1523， Rv3510c， Rv0867c， Rv3922c， Rv0722，Rv0733，Rv0721，Rv0709，Rv0546c，Rv0445c…	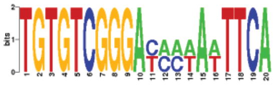
M23	12	Rv0327c，Rv3833，Rv3334，Rv0384c，Rv0325，Rv0324，Rv1049，Rv2963，Rv1767，Rv1766，Rv0326， Rv1048c	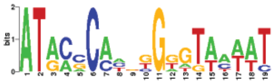
M24	13	Rv1658，Rv1653，Rv1652，Rv1655，Rv1654，Rv1657，Rv1656，Rv3677c，Rv3919c，Rv2937，Rv2731， Rv1857， Rv2939	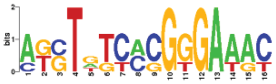
M25	26	Rv3373，Rv3135，Rv3101c，Rv1797，Rv0697，Rv0808， Rv0914c， Rv0256c， Rv3102c， Rv2428，Rv2429，Rv0270，Rv0377，Rv0870c，Rv3524，Rv2700，Rv3012c，Rv1796，Rv3901c，Rv3701c…	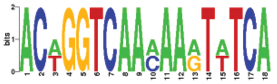
M26	115	Rv0938，Rv3741c，Rv3084，Rv3085，Rv1394c，Rv1869c， Rv3742c， Rv3086， Rv2909c， Rv2908c， Rv3460c， Rv1935c， Rv3740c， Rv3459c， Rv3458c， Rv0471c， Rv1328， Rv1245c， Rv3457c， Rv2918c…	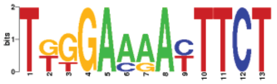
M27	73	Rv1901，Rv0596c，Rv2673，Rv2265，Rv2174，Rv0743c，Rv2564，Rv2238c，Rv0364，Rv3695，Rv1097c，Rv2291，Rv2173，Rv2229c，Rv2263，Rv0747， Rv1037c， Rv0961， Rv2611c， Rv3275c…	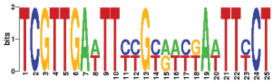
M28	53	Rv0022c， Rv3447c， Rv3448， Rv3905c， Rv3904c，Rv3911，Rv0026，Rv2079，Rv2078，Rv3439c， Rv3444c， Rv3445c， Rv3446c， Rv0031，Rv3449，Rv3906c，Rv0027，Rv1951c，	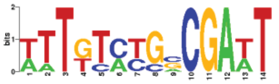

		Rv3453…	
M29	20	Rv1015c， Rv1621c， Rv0296c， Rv1620c， Rv1623c， Rv1622c， Rv1315， Rv2585c， Rv3116，Rv1483，Rv1296，Rv3482c，Rv1060，Rv0674，Rv1691，Rv1646，Rv2861c，Rv0128，Rv1298， Rv1295	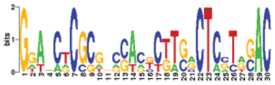
M30	14	Rv2822c， Rv2821c， Rv3857c， Rv1270c， Rv2819c， Rv2820c， Rv2880c， Rv3782， Rv1363c， Rv3335c， Rv2125， Rv2478c	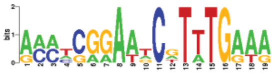
M31	28	Rv3534c，Rv3527，Rv3526，Rv3516，Rv3545c，Rv3538， Rv3537， Rv3535c， Rv3502c， Rv3515c， Rv3504， Rv3503c， Rv3569c， Rv3546， Rv3567c， Rv3574， Rv3916c， Rv3570c， Rv3573c， Rv2877c…	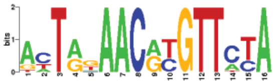
M32	20	Rv3563，Rv3552，Rv3565，Rv3564，Rv3518c，Rv3551，Rv3550，Rv3549c，Rv3562，Rv3556c，Rv3560c， Rv3557c， Rv3522， Rv0940c， Rv3559c， Rv3541c， Rv3506	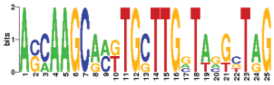
M33	15	Rv0019c， Rv0597c， Rv0819， Rv0182c， Rv2833c，Rv2422，Rv2416c，Rv0060，Rv1496，Rv0556，Rv0489，Rv0025，Rv2886c，Rv2816c，Rv0809	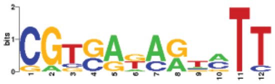
M34	54	Rv1634，Rv0018c，Rv2335，Rv0409，Rv0528，Rv0861c， Rv0527， Rv3092c， Rv2360c， Rv0542c， Rv0408， Rv2314c， Rv3220c， Rv2152c，Rv1422，Rv1095，Rv1480，Rv3605c，Rv1200， Rv2185c…	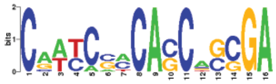
M35	28	Rv3402c， Rv2386c， Rv2383c， Rv2382c， Rv2123， Rv2381c， Rv2380c， Rv2379c， Rv1348，Rv1349，Rv3839，Rv3403c，Rv2378c，Rv1347c，Rv1519，Rv3841，Rv3840，Rv2377c，Rv2107， Rv1346…	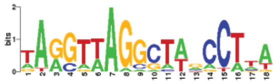
M36	51	Rv1022，Rv0986，Rv2295，Rv0891c，Rv2683，Rv2811，Rv3750c，Rv0187，Rv0001，Rv2684，Rv3789， Rv1516c， Rv1036c， Rv0398c， Rv1527c， Rv0873， Rv0971c， Rv3843c， Rv0087， Rv3819…	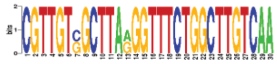
M37	21	Rv2941，Rv0253，Rv3344c，Rv2097c，Rv1925，Rv3743c，Rv0252，Rv1521，Rv1156，Rv0634c，Rv2200c， Rv1448c， Rv3286c， Rv0958， Rv2884， Rv1189， Rv3416， Rv2748c…	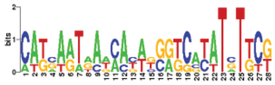

M38	15	Rv2126c， Rv3508， Rv3512， Rv3895c，Rv2770c，Rv2702，Rv2484c，Rv0415，Rv0416，Rv2490c，Rv1209，Rv2862c，Rv3364c，Rv3771c	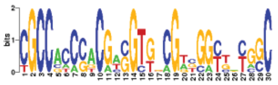
M39	29	Rv1991c，Rv3753c，Rv2196，Rv2194，Rv2195，Rv1872c， Rv2137c， Rv2147c， Rv2772c，Rv3301c，Rv2284，Rv3689，Rv2982c，Rv3008，Rv1549，Rv0442c，Rv3415c，Rv1293，Rv0452，Rv2155c…	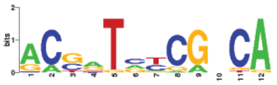
M40	50	Rv2566，Rv1134，Rv1900c，Rv1830，Rv0371c，Rv0582，Rv2592c，Rv2567，Rv0737，Rv2565，Rv1890c， Rv1888c， Rv2568c， Rv2545，Rv2505c，Rv2546，Rv0751c，Rv2521，Rv2233，Rv0521c…	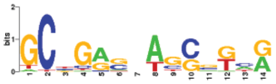
M41	79	Rv3155，Rv0173，Rv0174，Rv0172，Rv0170，Rv3158，Rv3149，Rv0168，Rv1312，Rv3157，Rv3154，Rv0144，Rv0178，Rv3145，Rv3153，Rv0169，Rv3152，Rv3156，Rv2773c，Rv0166…	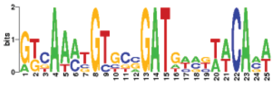
M42	21	Rv3755c， Rv2427c， Rv1583c， Rv1261c，Rv2426c， Rv3719， Rv3305c， Rv3455c，Rv2067c， Rv1084， Rv0257c， Rv3577，Rv1232c， Rv3779， Rv0507， Rv3749c，Rv0937c， Rv3682， Rv3576， Rv1167c…	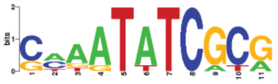
M43	18	Rv2620c， Rv1462， Rv1465， Rv2621c，Rv1685c，Rv1466，Rv1686c，Rv1463，Rv1464，Rv1687c， Rv1016c， Rv2619c， Rv2622，Rv0265c，Rv0346c，Rv2716，Rv2681，Rv3683	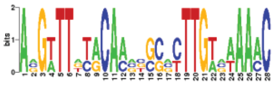
M44	15	Rv3713，Rv0730，Rv1532c，Rv2911，Rv3688c，Rv1860， Rv3052c， Rv2254c， Rv2656c，Rv0357c， Rv2270， Rv1498c， Rv1174c，Rv2469c， Rv2146c	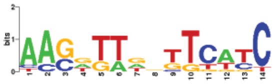
M45	31	Rv1697， Rv2350c， Rv2864c， Rv3005c，Rv1987， Rv2074， Rv0519c， Rv3587c，Rv3258c， Rv3414c， Rv2349c， Rv2119，Rv3209， Rv1541c， Rv0040c， Rv1157c，Rv3717， Rv1566c， Rv0063， Rv1220c…	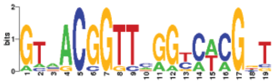
M46	55	Rv2299c，Rv2271，Rv3575c，Rv3747，Rv2582，Rv2272，Rv2061c，Rv2276，Rv2795c，Rv3748，Rv2275，Rv2959c，Rv2069，Rv2421c，Rv3218，Rv2520c， Rv0429c， Rv1980c， Rv2239c，Rv2211c…	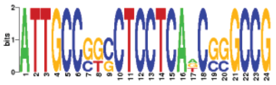
M47	21	Rv3615c， Rv3614c， Rv3616c， Rv3613c，Rv3823c，Rv1180，Rv1181，Rv1891，Rv3612c，Rv3825c， Rv1183， Rv2482c， Rv3824c，Rv1892， Rv1893， Rv3514…	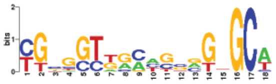

M48	43	Rv1324，Rv0014c，Rv0931c，Rv2936，Rv1121，Rv1747，Rv2905，Rv0545c，Rv2934，Rv3244c，Rv0543c，Rv2260，Rv2935，Rv2949c，Rv2933，Rv0133，Rv2948c，Rv2938，Rv0017c，Rv0555…	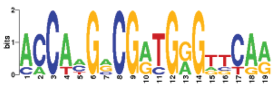
M49	15	Rv0704，Rv0720，Rv0719，Rv0708，Rv0707，Rv0723，Rv1704c，Rv0705，Rv0227c，Rv2403c，Rv0706，Rv2281，Rv0668，Rv3660c，Rv0667	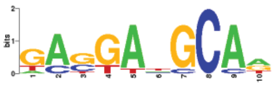
M50	32	Rv1306，Rv1438，Rv1305，Rv3775，Rv2476c，Rv1437，Rv2193，Rv3896c，Rv0831c，Rv3273，Rv3281，Rv2586c，Rv0509，Rv0896，Rv0470c，Rv1436，Rv1140，Rv1304，Rv3874，Rv1543…	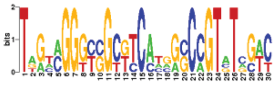
M51	37	Rv1107c，Rv1694，Rv0544c，Rv0177，Rv0936，Rv1614，Rv1613，Rv1806，Rv1710，Rv1711，Rv3146，Rv0073，Rv0694，Rv1427c，Rv1547，Rv1713，Rv3494c，Rv2050，Rv2127，Rv0890c…	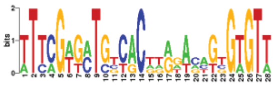
M52	22	Rv1108c，Rv0514，Rv0513，Rv1570，Rv3247c，Rv3212，Rv0512，Rv0526，Rv0866，Rv1573，Rv0138，Rv0042c，Rv0511，Rv0134，Rv0024，Rv1979c，Rv2903c，Rv3354，Rv1915，Rv1878…	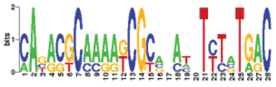
M53	80	Rv1003，Rv1011，Rv0613c，Rv0954，Rv0803，Rv0952，Rv1056，Rv0517，Rv3100c，Rv1002c，Rv1090，Rv0058，Rv2508c，Rv3384c，Rv0321，Rv2023c，Rv0728c，Rv0499，Rv0269c，Rv3076…	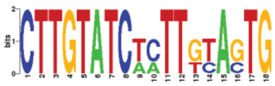
M54	16	Rv2273，Rv2563，Rv3834c，Rv0624，Rv2953，Rv3761c，Rv2066，Rv2523c，Rv2561，Rv2370c，Rv2186c，Rv1896c，Rv2807，Rv1231c，Rv0646c，Rv3271c	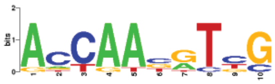
M55	34	Rv0221，Rv1870c，Rv2556c，Rv1841c，Rv1714，Rv3485c，Rv0823c，Rv2553c，Rv1871c，Rv2901c，Rv1717，Rv3284，Rv2364c，Rv2848c，Rv2552c，Rv0487，Rv1835c，Rv0135c，Rv2365c，Rv1715…	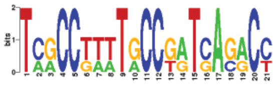
M56	24	Rv3821，Rv1309，Rv0575c，Rv0767c，Rv1307，Rv1311，Rv1708，Rv3648c，Rv1310，Rv1133c，Rv2241，Rv0124，Rv1838c，Rv1677，Rv1308，Rv1375，Rv0188，Rv2753c，Rv3592，Rv1676…	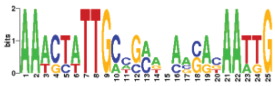
M57	11	Rv0447c，Rv0353，Rv0352，Rv0351，Rv0350，Rv3686c，Rv1461，Rv1094，Rv3418c，Rv0824c，Rv0348	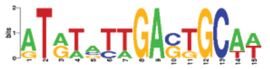

M58	77	Rv0982，Rv0589，Rv0611c，Rv0621，Rv1119c，Rv0664，Rv0627，Rv0623，Rv2286c，Rv0609，Rv0665，Rv0610c，Rv3386，Rv0607，Rv1741，Rv0605，Rv0738，Rv2613c，Rv3708c，Rv0626…	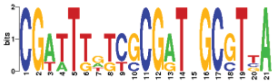
M59	88	Rv1737c，Rv0574c，Rv0572c，Rv2624c，Rv0570，Rv3133c，Rv2003c，Rv2628，Rv2004c，Rv2005c，Rv1997，Rv2029c，Rv2006，Rv3130c，Rv2032，Rv2030c，Rv1996，Rv3134c，Rv3129，Rv2031c…	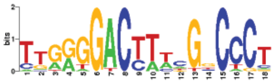
M60	50	Rv2828c，Rv2734，Rv0486，Rv2825c，Rv3846，Rv2665，Rv1178，Rv3628，Rv3820c，Rv2001，Rv3043c，Rv0478，Rv3528c，Rv1071c，Rv1649，Rv2329c，Rv0191，Rv0565c，Rv3318，Rv2215…	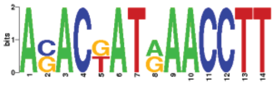
M61	41	Rv1569，Rv0999，Rv1044，Rv0435c，Rv0604，Rv3032，Rv0103c，Rv1340，Rv3264c，Rv1571，Rv0785，Rv3192，Rv1659，Rv2712c，Rv1263，Rv0076c，Rv0955，Rv3539，Rv0783c，Rv0183…	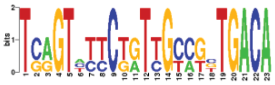
M62	61	Rv1584c，Rv1601，Rv3692，Rv3671c，Rv3451，Rv0197，Rv2228c，Rv3763，Rv2373c，Rv3610c，Rv3497c，Rv3493c，Rv2409c，Rv2156c，Rv3042c，Rv3362c，Rv1230c，Rv0420c，Rv3591c，Rv3413c…	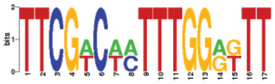
M63	22	Rv0717，Rv0718，Rv0716，Rv0714，Rv0651，Rv0715，Rv0703，Rv0652，Rv3442c，Rv3443c，Rv0640，Rv0710，Rv0701，Rv1488，Rv3029c，Rv0641，Rv3456c，Rv0639，Rv2115c，Rv1914c…	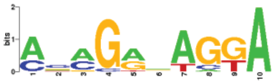
M64	35	Rv0749，Rv2662，Rv0366c，Rv1358，Rv2663，Rv1085c，Rv1406，Rv2177c，Rv0963c，Rv1530，Rv2664，Rv1419，Rv3424c，Rv3319，Rv3830c，Rv1246c，Rv1946c，Rv3629c，Rv0802c，Rv2249c…	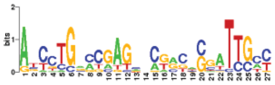
M65	41	Rv0074，Rv1679，Rv0995，Rv3722c，Rv0807，Rv1967，Rv2971，Rv0673，Rv3400，Rv1446c，Rv2430c，Rv0107c，Rv1109c，Rv3425，Rv1678，Rv0682，Rv3299c，Rv1681，Rv0928，Rv3697c…	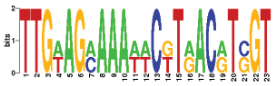
M66	16	Rv3837c，Rv2052c，Rv1086，Rv0251c，Rv2694c，Rv2374c，Rv2053c，Rv2745c，Rv2744c，Rv2372c，Rv2743c，Rv1072，Rv2710，Rv2699c，Rv3179	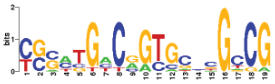

M67	17	Rv0286，Rv3020c，Rv0287，Rv0285，Rv0284，Rv3019c，Rv0289，Rv0288，Rv0292，Rv0290，Rv1664，Rv1719，Rv0283，Rv2849c，Rv3022c，Rv0282， Rv0666	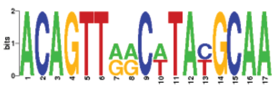
M68	34	Rv0299，Rv0005，Rv2009，Rv2110c，Rv2109c，Rv1415，Rv1595，Rv2010，Rv3609c，Rv1294，Rv0904c， Rv1594， Rv3256c， Rv0403c，Rv2064，Rv2021c，Rv2841c，Rv3327，Rv0518，Rv0006…	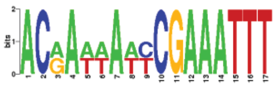
M69	39	Rv2118c， Rv1137c， Rv1291c， Rv0656c，Rv1802， Rv0657c， Rv2162c， Rv1728c，Rv2418c， Rv2359， Rv0137c， Rv2845c，Rv3650，Rv3059，Rv2447c，Rv2794c，Rv3736，Rv0150c， Rv1700， Rv1500…	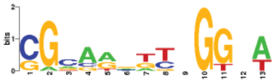
M70	63	Rv0315，Rv1492，Rv1493，Rv1826，Rv3791，Rv0145，Rv3030，Rv1219c，Rv2499c，Rv1057，Rv0504c， Rv2951c， Rv0271c， Rv1885c，Rv3808c，Rv0247c，Rv2692，Rv0308，Rv0333，Rv2996c…	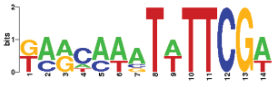
M71	24	Rv2637，Rv0401，Rv2438c，Rv2930，Rv0577，Rv3392c，Rv3302c，Rv2980，Rv0801，Rv2831，Rv3484，Rv1609，Rv0039c，Rv1253，Rv0538，Rv1561，Rv2809，Rv3211，Rv0413，Rv2431c…	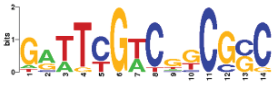
M72	24	Rv2778c， Rv2954c， Rv3499c， Rv0933，Rv2945c， Rv2128， Rv2829c， Rv2952，Rv1624c， Rv1771， Rv2992c， Rv3232c，Rv3234c，Rv0858c，Rv1152，Rv1952，Rv1746，Rv2475c， Rv2947c…	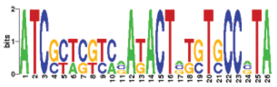
M73	325	Rv3066， Rv3161c， Rv0830， Rv0560c，Rv2651c，Rv3065，Rv1577c，Rv3183，Rv3872，Rv3406， Rv0677c， Rv0726c， Rv1578c，Rv1982c， Rv2650c， Rv2826c， Rv2872，Rv3160c， Rv0678， Rv2502c…	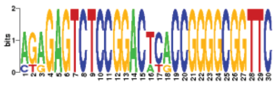
M74	37	Rv0869c， Rv3297， Rv0002， Rv2728c，Rv2135c， Rv2131c， Rv1725c， Rv0724，Rv2002，Rv2308，Rv1846c，Rv2682c，Rv3785，Rv1172c， Rv0821c， Rv1063c， Rv2133c，Rv1862， Rv0644c， Rv0273c…	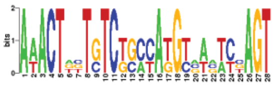
M75	42	Rv2528c， Rv1834， Rv2533c， Rv2530c，Rv1788，Rv2531c，Rv1531，Rv2527，Rv0213c，Rv2534c， Rv2250c， Rv1909c， Rv2529，Rv0970， Rv1829， Rv2535c， Rv1912c，Rv1833c， Rv2251， Rv3436c…	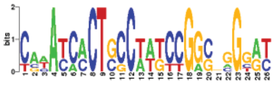

M76	143	Rv0474，Rv0950c，Rv1078，Rv2140c，Rv3292，Rv1650，Rv3011c，Rv3060c，Rv3855，Rv1456c，Rv2468c，Rv3173c，Rv0064，Rv3712，Rv0479c，Rv0957，Rv1886c，Rv2294，Rv3794，Rv0760c…	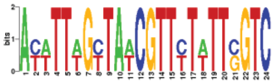
M77	17	Rv3479，Rv0165c，Rv0211，Rv0158，Rv0696，Rv3295，Rv0788，Rv3705c，Rv0695，Rv2485c，Rv2486，Rv1368，Rv2181，Rv2740，Rv0457c，Rv0468，Rv1171	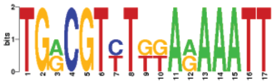
M78	35	Rv2571c，Rv2572c，Rv2142c，Rv3340，Rv3274c，Rv1852，Rv1475c，Rv3015c，Rv2569c，Rv2609c，Rv0268c，Rv3068c，Rv2197c，Rv0011c，Rv0206c，Rv3369，Rv0581，Rv2395，Rv0212c，Rv1023…	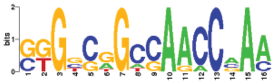

The first column is module number, named after the color, whose order is：antiquewhite4，bisque4，black，blue，blue2，brown，brown2，brown4，coral1，coral2，cyan，darkgreen，darkgrey，darkmagenta，darkolivegreen，darkolivegreen4，darkorange，darkorange2，darkred，darkseagreen4，darkslateblue，darkturquoise，darkviolet，firebrick4，floralwhite，green，greenyellow，grey60，honeydew1，indianred4，ivory，lavenderblush3，lightcoral，lightcyan，lightcyan1，lightgreen，lightpink4，lightsteelblue，lightsteelblue1，lightyellow，magenta，maroon，mediumorchid，mediumpurple2，mediumpurple3，midnightblue，navajowhite2，orange，orangered3，orangered4，paleturquoise，palevioletred3，pink，plum，plum1，plum2，plum3，purple，red，royalblue，saddlebrown，salmon，salmon4，sienna3，skyblue，skyblue1，skyblue2，skyblue3，steelblue，tan，thistle1，thistle2，turquoise，violet，white，yellow，yellow4，yellowgreen.
